# Regenerative medicine: Stroke survivor and carer views and motivations towards a proposed stem cell clinical trial using placebo neurosurgery

**DOI:** 10.1111/hex.12632

**Published:** 2017-10-12

**Authors:** Nicola A. Cunningham, Purva Abhyankar, Julie Cowie, Jayne Galinsky, Karen Methven

**Affiliations:** ^1^ University of Stirling Stirling UK

**Keywords:** caregiver burden, consent, regenerative medicine, treatment decision‐making

## Abstract

**Background:**

Few studies explore stroke survivor views and motivations towards stem cell therapy (SCT). This qualitative study explores the views and motivations of both stroke survivors and their partners/carers towards a proposed 2‐arm Phase III Randomised Controlled Trial (RCT) comparing intracerebral insertion of stem cells with placebo neurosurgery in stroke survivors with disability.

**Objective:**

To explore views and motivations towards a proposed 2‐arm stem cell trial and identify factors that may impede and enhance participation.

**Design:**

This study adopts a naturalistic design to explore the complexity of this field, employing a participatory action‐research approach comprising a specialized Conversation (World) Café form of focus group. Data were collected via 5 Conversation Cafés with stroke survivors (age 40‐75) and partners/carers between June and October 2016. Of 66 participants, 53 (31 male, 22 female) were stroke survivors and 13 (6 female, 7 male) were partners/carers. Qualitative data were analysed using a thematic approach.

**Discussion and Conclusion:**

Stroke survivor views and motivations reflect anticipation of the personal and future benefits of regenerative medicine. Partners/carers sought to balance the value of stroke survivor hope with carrying the weight of hope as carer, a conflict burden adding to known caregiver burden. All participants expressed the need for during and post‐trial psychological support. This study provides a rare opportunity to explore the prospective views and motivations of stroke survivors and their partners/carers towards a proposed Phase III 2‐arm RCT. This adds weight to qualitative evidence exploring capacity, consent, decision making, perceptions of treatment risk and supports required for clinical trial participation.

## 1 INTRODUCTION

Stroke is the third leading cause of complex disability: over half of 1.2 million stroke survivors live with disability caused by damage to brain tissue/cells as a result of blockage or haemorrhage of the blood supply.^1^ Stroke can impair a range of cognitive and physical functions and significantly impact on individuals’ and carers’ quality of life.

Recent scientific research focuses on investigating the therapeutic value of stem cells to replace lost or damaged cells from stroke. Early stage phases I and II clinical trials have demonstrated the safety of injecting intracerebral stem cells.^2^, ^3^ The proposed Phase III Randomised Controlled Trial (RCT) aims to assess the safety, tolerability and efficacy of intracerebral stem cells in patients with disability following an ischaemic stroke. Participants will be randomized (2:1) to active or placebo neurosurgery. Stereotaxic methods will be used to inject the cells via a cannula into the damaged hemisphere using local or general anaesthetic and analgesia. For patients allocated to receive placebo, only the outer table will be removed and the dura will not be breached during the procedure. Similar placebo‐controlled surgical trials have been conducted in other fields (in Parkinson's disease, eg^4^, ^5^), but the proposed stem cell therapy RCT is novel in the field of stroke and has the potential to raise questions from participants and the medical community.

Many trials struggle to recruit and retain target patient numbers, and only about 50% reach their recruitment target.^6^, ^7^ This jeopardizes the trial's internal and external validity, potentially delaying the development of more effective treatments.^8^, ^9^ Recruitment is reported to be particularly difficult for trials involving randomization and placebo arms^10^, ^11^, ^12^ and is predicted to be challenging for trials involving stem cells due to anticipated ethical and moral uncertainties about risk‐benefits.^13^ Among the factors influencing trial recruitment rates, patients’ beliefs and preferences have been identified as key barriers to participation.^14^ This study provides further evidence highlighting the views and motivations of the target patient group and identifies factors that may impede and motivate patient participation.^15^

Ethical principles governing clinical trials require that patients provide informed consent following a voluntary and informed decision about taking part or not.^16^ An informed decision requires that individuals consider information about all available options and their consequences, evaluate this information in accordance with their own values, preferences and circumstances and make a deliberate choice based on trade‐offs between these evaluations.^17^ In the context of stem cell trials for stroke, this process is also likely to be shared among patients, carers and their health professionals, each bringing their own values and preferences.^18^ Factors such as contrasting views and motivations, expectations of curative benefits, and the context in which trial recruitment takes place may render the decision‐making process susceptible to biases.^19^, ^20^ Recruitment procedures therefore must ensure that patients and families are enabled to make an informed decision. Study sponsors must balance the ethical aspects of the Phase III trial protocol and potential therapeutic misconceptions against strategies to optimize trial recruitment. Standard processes put in place to enable informed consent have been recently criticized as suboptimal for not meeting the needs of patients in terms of making an informed decision, calling for alternative approaches to be developed.^20^, ^21^, ^22^, ^23^

Given the dual challenge of maximizing trial recruitment while respecting and supporting individuals’ informed decision making, this research was sponsored to further understand the views and motivations of people affected by stroke and their partners/carers towards the proposed Phase III two‐arm trial. While current evidence^13^, ^24^ highlights some positive motivations towards stem cell therapy, this study extends understanding by highlighting stroke survivor and partner/carer views as a two‐way involvement process, revealing key impediments that require targeted support to enhance participation in the proposed Phase III trial.

## 2 METHODS

### 2.1 Design

Qualitative methods are well suited for investigating concepts such as views and motivations within the patient community to help explain the processes at work in the uptake of new interventions and decision making around trial participation.^25^

This study adopts a naturalistic design^26^ to explore complexity in this field, adapting from a participatory action‐research approach comprising a specialized World (Conversation) Café form of focus group. The Café approach creates a relaxed, informal and conversational environment, facilitating constructive engagement around complex issues and critical questions. This is an effective format for hosting interactive and large group dialogue. Each Conversation Café event begins with an introduction by the lead host (first author) to set the context and put participants at ease. Each event comprises up to five tables (4‐5 participants) and one host (researcher) and consists of a series of conversation topic rounds lasting up to 20 minutes (Table 1). The role of each table host is to provide structure and orienteering as the café process unfolds, encouraging conversation without taking over. A final plenary session, delivered by the lead host, draws together key agreed themes, inviting individuals to share further insights from their larger group conversations.^27^

**Table 1:** Conversation Café rounds

Round 1	Proposed trial: all views
Round 2	Information needs
Round 3	Trial protocol: skewed randomization and placebo group
Round 4	0‐12 months post‐stroke

### 2.2 Population, sampling and participant recruitment

Potential participants were identified from UK stroke community support groups. These groups can be advantageous to stroke survivors and their partners/carers as they often combine social function with physical rehabilitation therapy. While each individual stroke experience may be unique, there are common themes within stroke narratives and many use the support group to share their strategies for overcoming challenges.^28^

Sampling decisions were conceptually driven.^29^ At the centre of this framework were the experiences of stroke survivors and their partners/carers. Purposive predetermined sampling procedures were used to identify community groups based on relevance to the research topic and to ensure a broad range of views and motivations.^30^ Sample size was guided by early analyses taking place alongside continuing data collection, until no new themes emerged from the data and data saturation could be confirmed.^31^

### 2.3 Data collection

Data were collected via five Conversation Café events held June and October 2016. Criteria for participation were stroke survivors aged 40‐75 who had experienced stroke within the previous 36 months and partners/carers. Of the total 66 participants, 53 (31 male and 22 female) were stroke survivors; four of whom had experienced recent stroke within 12 months and 13 (six female, seven male) were carers. While this research sample is not representative of the diversity of the general population, Café locale represent a mixed demographic from across the UK.

### 2.4 Materials

Information sheets, consent forms and topic guides were provided prior to the Conversation Café event at scheduled pre‐Café information sessions (led by first author) and during Café registration if required. Participants were only recruited to the study if they could give informed consent. This meant that participants had a clear understanding of what involvement in this research meant and what was expected of them. All participants were advised that participation was voluntary and did not prejudice future treatment choices. If individuals were unable to sign consent forms, then verbal consent was accepted.

Conversation Café topic guides with probes (Table 2) were informed by evidence and literature on experience and attitude towards placebo‐controlled trial^31^, ^32^, ^33^, ^34^; the effect of randomization as a predictor of volunteering^35^, ^36^; therapeutic misconception,^37^ attitudes to stem cell use and donation.^38^, ^39^

**Table 2:** Conversation Café topic guide

What do you think about the proposed clinical trial? a What are your main concerns about trials like this?
How would you feel if your partner/relative was taking part?
What kind of information would you need to be able to make a decision about taking part in a trial like this?
Do you think any of the following would affect someone's decision to take part? a More likely to receive the actual treatment than be part of the control group (ie skewed participation) b Or if part of control group be offered the same treatment (if successful) at a later date/12 months? c Would you prefer to be asked what preference you have? That is, prefer to receive the specific treatment; prefer not to receive treatment; or are neutral?
Thinking back to the first 12 months after diagnosis, would your opinion have been different in any way?
Is there anything else that would affect your decision to take part?
If you decided to take part, what would be your reasons?
If you decided not to take part, what would be your reasons?

Each table, with refreshments provided by the research group, held a focused group conversation using the study topic guide.^40^ All Conversation Café events were audio‐recorded and transcribed verbatim. Participants were also provided with Post‐Its, pens/pencils and writable tablecloths to record any notes or comments. This was also employed as an aid for those with speech difficulties or reluctant to voice opinion within a group setting.

### 2.5 Ethical considerations

All Conversation Café hosts were skilled qualitative researchers, experienced conducting research with vulnerable groups and around sensitive issues. This study received ethical approval from the University Research Ethics Committee (version 2: 07‐04‐16).

### 2.6 Analysis

**Figure 1:**
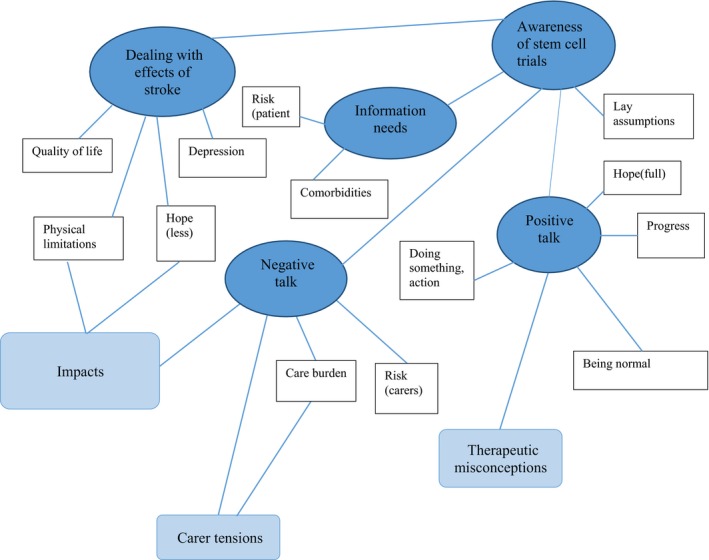
First organizing themes: 5 main themes

**Figure 2:**
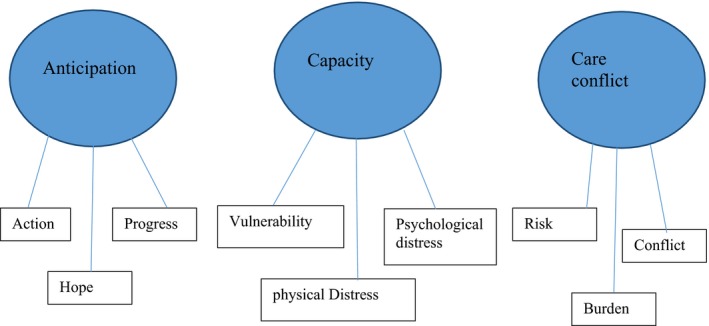
Global Schematic: 3 main themes

Data were analysed using thematic analysis^41^ to identify, interpret and report patterns and themes within the data. An initial coding framework (Table 3) was developed with data from 2 Conversation Café's, chosen to provide a fair representation of the range of participants. A constant comparative process comparing the relative frequencies of theme and topic enabled initial coding of data. Box 1 provides example of coded data extracts alongside early interpretive processes. Two members of the research team undertook this procedure simultaneously. Similarities and consistencies ensured reliability and trustworthiness of the analytical process. Table 4 provides example of the movement from the initial coding framework to the next stage mid‐ordering of codes. The codes illustrated provide example of diversity and pattern within the data. Those too diverse or lacking in depth were discarded. The next analytic stage focused on searching for themes and combining different (and sometimes similar) codes to represent aspects within the data. Visual thematic mapping enabled the consideration of links and relationships to support the interpretive process. Figure 1 illustrates the movement between the mid‐stage ordering of codes to first organizing themes. This reflects a process of clustering, collapsing and combining codes that shared unifying features so that they reflected and described meaningful patterns. For example, we noticed code clustering and overlap around risk, motivation and caring. Examined in detail, we identified the codes reflected positive and negative talking and often conflicting views and motivations between patients and partners/carers. Figure 2: Global Schematic represents a visual thematic map illustrating the final process of defining, refining and naming themes to capture the most important and relevant elements of the data. Box 2 clearly states what is unique and specific about each theme.

**Table 3:** Initial coding framework

Information needs
Placebo and skewed randomization
Anaesthetic risk
Physical capacity
Depression (known and unknown)
Quality of life
Carers support needs
Carer risk and loss
Lay knowledge and assumptions
Altruistic motivation
Scientific progress
Lay knowledge/therapeutic misconceptions
Psychological effects/concerns
Patient vulnerability
Being normal
Comorbidities
Hope(full)
Hope(less)

Box 1 **Example coded data**

1

**Table:** 

Data extract	Coded for
*So you would support him in this type of trial?* (Café Table Host) *Yes but like I said, he needs hope but maybe I don't* [pause]. *He would be full of it. You know what he's like? He'd convince himself every bit of movement was some change because of it. And maybe it might be but maybe not?* [Pause] *It's so difficult* [E: C50) *Would anything help you?* (Café Table Host) *I don't really know. Someone to talk to but I don't know really? I don't have much time. Not sure if I'm into that really! I'm not sure if talking about one bit would be like taking my finger out of the dam* [pause] … [laughing] … *If I say no it feels like I'm condemning him*. (E: C50)	Carer risk, loss and support needs. Managing carer burden Managing hope (patient and carer perspectives) Patient vulnerability enhanced by anticipation Therapeutic misconceptions affected by lay assumptions
*… We need to do this but I'd worry like? Worry for her in case anything went wrong. I do most of the care and look after the kids. So does our parents … I don't know if they'd understand; cope with more worry. I wouldn't want to give them any hope? They've been so upset. But I know she'd want this, wouldn't you* [looking at E: P2) P2 nodding] [pause] *But* [pause] *it would be hard to go through this and see it not work*. (E: C2)	Risk Carers worry about coping and burden Carer and patient vulnerability Feeling conflicted: two‐way decision making Hope(full)
*So, Superman was right wasn't he?* [group nodding strongly in agreement] *Who'd have thought?” Shame he couldn't last longer. Tragic. They could've had him up and walking by now, eh?* [group nodding in agreement] (E: P5) Table Host: *I'm not sure any improvement would be as dramatic as that? Does that make a difference?* *I know you don't want to give us too much hope but I think there's a lot going on isn't there? Ah mean, it hasn't been that long since Superman. Now it's Michael Schumacher, isn't it? Next thing we'll see is him walking, y'know? That's why there's a news blackout on it*. (E: P5) [group nodding strongly in agreement]. Table Host: *I'm not so sure. Do you really think so?* ***Mark my words*** [emphasis] *and watch for the papers!* [E: P5]	Therapeutic misconceptions lay assumptions and media effects Too much hope(full)? Risk‐averse? Patient vulnerability linked to assumptions Anticipation and the superman effect

**Table 4:** Mid‐ordering: 7 codes with illustrative data extracts

Motivations affected by lay assumptions	Trial information needs	Risky decision making	Early decision making post‐stroke	Post‐trial experiences and supports	The effects of hope	Carers involvement and conflict
… And would it bring things back? Obviously the things I've forgotten, Y'know? So superman was right wasn't he? … They could've had him up and walking by now	… you'd want to know the surgeon was professional … and that they knew what they were doing … and if they knew that I'd lost my speech … I'd be a bit worried about stuff. Like do they screen them? Make sure they're safe? I want to know I have a fair chance of getting treatment …	So you're saying then that I won't know whether I've been given them … and I might get the change to have them later? So that would be two anesthetics then? They wouldn't allow me to fly for a year because they were scared of a bleed… so going back to 3‐11 months? It's part of the medical intervention … you must take it easy … There's a bit of confusion there?	… But if I'd been asked to participate in that first few months, I would've said yes but I might've been confused a bit … It took me a whole for the fog to clear … If you'd gone to ask her within the first year … it's cognitive … you wouldn't have got a rational answer …	What I'm thinking is you can be set in your ways after stroke … And if you find out it's placebo like … you might get really down‐hearted. What happens to everyone after the trail has ended? Will anyone check how we're doing? You'd have to make sure people coming out are getting extra help …	… It might give hope … Y'know false hope to some people … how would they cope with that? I think they would need an awful lot of support to come to terms with that afterwards? … Yes but like I said he needs hope but maybe I don't? Anything … **any change** [emphasis]. Some **hope** [emphasis] … would be worth it	… a lot of changes appear cognitively, memory … … so I do think as well like it needs the carers consent? *It's often harder for the partner [pause] …watching on, from the outside, for their partner and in any situation like that looking on,* She was still in shock. Couldn't hold the kids? She'd have jumped at it …

Box 2 **Definitions and labels**

**Anticipation: Superman was right, wasn't he?**

Outlines how stem cell therapy is a fast‐developing area of research and treatment, driven by better understanding of potential therapeutic opportunities and promising early phase clinical trials. Participant narratives are influenced by strong media coverage exalting the recovery of people disabled by stroke often enhancing known therapeutic misconception. Participants—particularly stroke survivors—praised the potential assumed benefits via stem cell therapy—and in contrast to the measured information given to them. Generally, positive media reporting also appeared to influence the general acceptability of participation in stem cell trials. While some participants requested further detail, most expressed a lack of concern. In general, participants—stroke survivors in particular—held strongly positive optimism towards stem cells which may require specific and targeted support.

**Capacity and decision making: Making a snap decision**

Outlines how participants identified key issues in relation to capacity and vulnerability during the proposed trial decision making and treatment period. Participants highlighted the difficulties of known cognitive, orientation and functional impairments post‐stroke and expressed concern around capacity for informed consent during the time‐period proposed. Many also highlighted the unknown difficulties that may emerge during and beyond participation.

**Care conflict: Balancing hope**

Focuses on developing understanding of known caregiver burden^54^, ^55^ and in relation to participation in clinical trials. This third theme also represents elements of the above 1st and 2nd final themes. Negative and positive discussions around the proposed trial reflected conflicting motivations and competing pressures. Partners and carers narratives illustrate a two‐way involvement in trial participation decision making, constantly weighing up perceived risks and benefits and assuming some responsibility for managing motivations. While all partners and carers provided support, the strong notes of caution and concern constitute important additional elements in our understanding of caregiver burden, trial decision making and the extended supportive interventions required during trial recruitment, participation and post‐participation.

## 3 RESULTS

The themes identified below represent themes identified, albeit some elements may overlap. This does, however, represent relationships between views and motivations as opposed to suggesting all concepts should be understood in isolation. The coding key used reflects Café (A‐E), P or C (patient or partner/carer) and numerical participant identifier.

### 3.1 Anticipation and regenerative medicine: Superman was right, wasn't he?

Anticipation of possible therapeutic benefits enhanced stroke survivor's positive motivation towards the proposed trial. The suddenness of stroke can leave the individual with a sense of discontinuity^42^ combined with feelings of loss, fear and hopelessness. Views and motivations towards participation connected to feelings of hopelessness and the lack of extended post‐stroke rehabilitation services and support to aid a hoped‐for “return to normal” (A: P6). Many participants reported feeling left to their own fate by the health service after only short periods of rehabilitation. This, combined with pervading disability and reduced quality of life, informed some strong motivation towards the proposed trial:

Anything [pause] **any change** [emphasis] [pause] would be worth it.

(E: P2, severe aphasia)

C: P1 had experienced ischaemic stroke after anaesthesia complication during a routine and scheduled operation:

… I thought I was indestructible [pause] **indestructible** [emphasis] but I would do it if there was a chance. **Anything** [emphasis] is better than this!

(C: P1, aphasia)

As an emerging science, regenerative medicine is surrounded by many myths and misperceptions, evoking enthusiasm and passionate debate alongside heightened health expectations.^43^, ^44^ Stroke survivor views and motivations reflect less the measured risk‐benefit evaluation and more strong anticipation of personal and future benefit combined with scientific breakthrough:

It's amazing what a stem cell can do to repair areas! Maybe in the future it can turn a face back 30 years, which would be good. I'll go for that!

(C: P1)

I know it might not be for me, for me ever … but to think that people are doing something [pause] that feels good! [Excitement] I'd like to replace Jessica Ennis at the next Olympics! [Group laughing] (E: P3) The partner of P3 noted this reaction: I haven't seen you light up like this for a while!

(E: C3)

Many stroke survivors overestimated possible benefits. This continued through all discussions, despite Table Host attempts to temper assumptions around therapeutic benefit. There was little evidence that the presentation of a more measured view of possible physical improvement had any impact on views and motivations towards this type of trial:

My dementia was caused by my stroke. Would this treatment sort my dementia as well? And would it bring things back? Obviously, the things I've forgotten, y'know?

(A: P2)

So, Superman was right wasn't he? [group nodding strongly in agreement] Who'd have thought?” Shame he couldn't last longer. Tragic. They could've had him up and walking by now, eh? [group nodding in agreement]

(E: P5)

Table Host: *I'm not sure any improvement would be as dramatic as that? Does that make a difference?*

I know you don't want to give us too much hope but I think there's a lot going on isn't there? Ah mean, it hasn't been that long since Superman. Now it's Michael Schumacher, isn't it? Next thing we'll see is him walking, y'know? That's why there's a news blackout on it.

(E: P5) [group nodding strongly in agreement]

Table Host: *I'm not so sure. Do you really think so?*

**Mark my words** [emphasis] and watch for the papers!

[E: P5]

Highly anticipative discussion can be contextualized against the sudden impact of stroke and often long‐term effects. Views and motivations were conditionally influenced by strong assumptions around therapeutic benefit and may extend beyond motivations towards the collective good. While these findings resonate with known work highlighting conditional altruistic motivation^43^, ^44^ towards trial participation, in this study, participation‐benefit‐risk reasoning was measured against at times unrealistic assumptions around treatment outcomes.

### 3.2 Capacity and decision making: A snap decision?

Participants were invited to reflect back to the 3‐ to 11‐month period post‐stroke during conversation. All groups raised concerns over the value of physical therapy and rehabilitation services. For many, these services had been restricted to 6 weeks post‐stroke. Many described a sense of abandonment by mainstream health services when this ended. The proposed trial offers assignation to a physical therapy programme pre‐randomization, to be self‐delivered after an initial training programme. Physical therapy would be continued for 12 weeks post‐surgery.

Concern around physical capacity affected positive motivation towards the proposed trial. Participants expressed some unease and described the incapacitating effects of stroke on cognition, orientation and functional capacity. Participants were advised that the proposed trial would include pre‐trial screening and cognitive/quality of life assessment (Rankin Scale). While this did enhance motivation towards trial participation, many participants stated the extent of impairment and vulnerability was only revealed retrospectively:

It's been 18 months since, now. Y'know, I'm not crying as much now [laughing] But I think if I'd been asked to participate in that first few months, I would've said yes but I might've been confused a bit … It took me a while for the fog to clear … I only knew I was in a fog when it was gone.

(E: P9)

Just to add to what we were saying—I know they'll assess you and see whether they think you could take part and you're ok an’ that [pause] But not many others really noticed that [looking at partner] I just seemed a bit quiet didn't I? [partner nodded] [pause] but I didn't know it was more than that but I don't know whether they could pick that up, y'know?

(E: P5)

Many participants viewed psychological support both during and beyond the trial as a critical support mechanism. This was because trial participation would combine with acute‐stage stroke recovery at a time when cognitive and functional improvement may be slow.

The invisible consequences of stroke often include uncontrolled emotion compounded by short‐term memory loss and problems with comprehension. Participation and decision must depend on participants understanding the aims, treatment, protocol, possible risks, benefits and rights to withdraw from trials.^45^ Reflecting back, some participants considered the immediate period post‐stroke as a time of “feeling vulnerable” (C: P3). Making decisions during this period was considered problematic:

It was a tough time. Can imagine it would have been difficult to think about it? I dunno [pause] maybe some would be [pause] vulnerable?

(E: P5)

For some, this increased the likelihood they would make a “snap” decision. Acute illness can impair understanding—and particularly concepts of proportionality and risk. Even when tests of cognitive function appear normal, there may be other unknown influencing factors and this reflects some concerns:

I probably would have grasped at straws, I don't know. I [pause] desperation? Yes, I was massively depressed and I would have been glad of perhaps anything that would have perhaps helped.

(E: P6)

… Actually, one of the things about the trial I worry about already is Group B [placebo]. They're going to face the possibility that they are in the wrong group. That needs a lot, I mean, the depression and the anti‐climax when they find out they didn't have anything [pause] the expectation of that I think would be devastating [pause] And then Group A. You'd need help for disappointment you know if it's not going to work [pause] helping you if you're thinking this is a cure type of thing you know?

(A: C1)

Given potential difficulties assimilating trial information during the acute post‐stroke period, participant transition through and beyond the trial was considering as troubling by participants:

Some people the depression hasn't come on for … 12 month… people think that when they've left [hospital] and come home, when they've got over that threshold of home there's fairy dust … kind of magic … that makes everything alright but it doesn't. There's a whole different set of problems; the depression can kick in, so this could be 6‐12 months down the line and they've already got involved in the research thinking it's the hope of all hope and then they're not getting miraculous results that they would expect regardless of what expectations people have said, that can make it worse I think.

(C: C1)

Participants described the trajectory towards depression as a slow one—a real fear for some and a reality for many. The difficulty negotiating this trajectory combined with trial participation while hoping for recovery was a strong concern. All participants identified the need for during and post‐trial psychological support.

### 3.3 Care conflict: Balancing hope

Caregiver burden is recognized as a significant health‐care concern^46^ and elevated for an indefinite period following stroke.^47^ Carers play an important role in post‐stroke rehabilitation, may enable a return to live at home or in the community and potentially positively impact on health outcomes. In this study, partner/carer views towards the proposed trial were qualified by key fears and concerns. Many first described feeling inadequately prepared for their unexpected role as carer. Although generally supportive of stem cell research and the proposed trial, partners/carers raised concerns around the potential risks of participating in this type of trial and the impact of regret if the trial did not bring individual benefit or caused harm:

I think if someone has had a stroke they really need to understand what has happened. And the person making the decision really needs to understand, and for the family too and consent and things [pause] My main concern would be that the operation could go wrong, I mean because every operation has a risk, I'd worry that it would make him worse.

(B: C2)

I'd be concerned about the fear of how it would affect you? [Looking at partner, C: P1]. How disappointed you would be if it didn't work as [C: P1] has depression which could get worse [pause] coping with the depression and the anti‐climax when they find out they didn't have anything?

[C: C1]

The prevalence of neurological impairment, depression and anxiety is known effects continuing up to 5 years after an incidence of stroke.^48^ Partner/carer views about the proposed trial also highlighted psychological concerns in relation to the possibility of post‐treatment regret, anticipated to bring additional difficulties not only to stroke survivors but also to partners/carers. Partners/carers reported watching “from the outside” [D: C1] and feeling conflicted between the possible hope offered by stem cell therapy, regret if difficulties were experienced and guilt:

It's often harder for the partner [pause]. Well, not harder, that's a lie, but somebody that's watching on, from the outside, for their partner and in any situation like that looking on, it's often harder, if the person doesn't realize what's happened to them. But we'd all like to benefit. I'll not say anymore [pause] I'm getting the look [from C: P1] C: C1 becomes emotional.

… We need to do this but I'd worry like? Worry for her in case anything went wrong. I do most of the care and look after the kids. So does our parents … I don't know if they'd understand; cope with more worry. I wouldn't want to give them any hope? They've been so upset. But I know she'd want this, wouldn't you [looking at E: P2, P2 nodding] [pause] But [pause] it would be hard to go through this and see it not work.

(E: C2)

In this study, a new dimension was revealed as partners/carers felt conflicted as they sought to balance the value of hope for the stroke survivor with carrying the weight of hope felt as carer.

The carer has to go through the fact that their loved one who's nearly died, who's had a stroke, it's a big thing [pause] but they've not died but they're going to put themselves at further risk with having a hole drilled [pause] and they might desperately want it because they think it's going to do them the world of good but the carer you know is on the end of that.

(E: C50)

This conflict adds to known caregiver burden and was sometimes expressed as guilt for not being supportive enough:

So you would support him in this type of trial?

(Café Table Host)

Yes but like I said, he needs hope but maybe I don't [pause]. He would be full of it. You know what he's like? He'd convince himself every bit of movement was some change because of it. And maybe it might be but maybe not? [Pause] It's so difficult

[E: C50)

Would anything help you?

(Café Table Host)

I don't really know. Someone to talk to but I don't know really? I don't have much time. Not sure if I'm into that really! I'm not sure if talking about one bit would be like taking my finger out of the dam [pause] … [laughing] … If I say no it feels like I'm condemning him.

(E: C50)

Across all groups, discussion revealed some key differences between stroke survivors and partners/carers. This provided further insight into the needs of stroke survivors and partners/carers and the support mechanisms that may be required to enhance participation.

## 4 DISCUSSION

This study provides a rare opportunity to explore the prospective views and motivations of stroke survivors and their partners/carers towards a proposed 2‐arm RCT using stem cells via placebo neurosurgery. Our findings add qualitative weight to recent evidence (see Ref. 24). The following section considers the factors that further impede participation and suggests strategies to enhance participation.

### 4.1 Conflict burden

It is important to highlight that supportive views and motivations towards the trial were influenced by high expectations of personal benefits, a “conditional altruism” influenced by (i) strong media reporting of earlier trial results and (ii) therapeutic misconception concerning the restorative potential of stem cells.^43^, ^49^ However, partners/carers revealed contrasting viewpoints and motivations. Partners/carers of survivors with neurological disease are known to have a higher risk of physical, mental and emotional depletion and reduced quality of life.^47^ Our findings indicate that partner/carer views and motivations were influenced by different perceptions and trade‐off between risk‐benefit. We introduce the concept “conflict burden”: carers report conflict, guilt and increased concern in relation to assumed physical and psychological risks involved when faced with stroke survivor's motivations towards clinical trial participation. This “conflict burden” was underlined via described tension between stroke survivor hopefulness and carers felt‐conflict supporting hopefulness.

### 4.2 Two‐way involvement

Stroke survivors and their partners/carers discussion highlighted a two‐way involvement in decision making. These findings remind us that when developing information and decision‐aids, survivor views should be placed at the core but this should not be to the exclusion of the views of partners/carers. Our study adds to evidence for trial information to be framed in the context of patients’ and partners/carers—interpersonal relationships and complex decision‐making processes.^22^, ^50^, ^51^

It is worth noting that some consideration has been given to this issue in related fields.^52^, ^53^, ^54^ Alzheimer's, as an irreversible neurological disease, also impairs cognition, orientation and functional capacity. The carer is often actively supported to be involved in decision making. Current recommendations to support participation in clinical trials position the carer as “study partner” to aid with decision making and consent.^55^ Stroke carers also experience the effects of stroke as sudden, unpredictable and disruptive, affording insufficient time for preparation in advance of complex care responsibilities. While carer involvement in decision making may relieve some of the difficulties of cognition, orientation and functional capacity, conversely, this responsibility may also cause conflict and add weight to known carer burden.^56^, ^57^ Our data suggest interventions should be mindful of the complex and at times, conflicting caring and interpersonal context^14^, ^18^, ^32^ when exploring ways of involving and supporting carers through the clinical trial decision‐making process.

The timing of the proposed trial during the 3‐ to 11‐month period post‐stroke was a concern for all participants, particularly in relation to the difficulties of survivor cognition, orientation and functional capacity. The acute effects of stroke may also include being prone to impulsive decision making and experiencing impairment balancing rewards with risk.^58^ This may combine with uncertainty, disruptive and shifting changes, a trajectory of post‐stroke depression, either in the early or in late stages after stroke. During this period, stroke survivors must evaluate the trial and make decisions about risk‐benefit. This represents a delicate trade‐off^59^ and one that may be compromised during the acute recovery period.

Our findings highlight key concerns and add evidence to recent work exploring decision making and strategies to alleviate recruitment difficulties.^20^, ^43^, ^60^ The conflicting effects of hope on stroke survivors and partners/carers are a notable concern. These effects may make trial participants more vulnerable and carers/partners more burdened.

### 4.3 Enhancing participation

The findings of this study have implications for supportive care through clinical trial participation. Recent developments have made positive moves forward. Current trialists are including evaluation of their recruitment strategies, a development received positively by Funding Councils and recommended via the UK Medical Research Council. Development includes the QuinTet Recruitment Intervention (QRI), evolved from^15^, ^60^ a pioneering embedded complex recruitment intervention in the ProtecT trial. The Q‐QAT intervention uses initial quantification of recruitment appointments to inform qualitative research to understand the issues and make suggestions to improve recruitment.

While many trials seek tools to inform and facilitate improved recruitment rates, there is less emphasis on requirements for post‐trial support. In our study, retrospective consideration revealed concerns around psychological support during and post‐trial participation; particularly, if the nature of impairment may escape detection during pre‐trial cognitive screening. For example, participants expressed concern their depression trajectory emerged during later months post‐stroke, and the possible negative impacts from trial participation, particularly unblinding to placebo post‐trial.

This study suggests strategies to enhance participation should include: consideration of consent as a two‐way involvement process between stroke survivors and carers, best obtained as (at least) a two‐stage process: initial consent followed by reaffirmed consent at interval and including appropriate time for reflection. Known and unknown cognitive and functional impairment and concerns around capacity and vulnerability were primary concerns in this study. Following NICE guidelines, this may be supported via the implementation of a stroke‐specific stepped‐care model of support at key interval periods. The support needs of partners/carers should be given independent consideration. Carer strain, burden and conflict should be reassessed regularly. This could coincide with trial follow‐up dates and be accompanied by direction to regularly scheduled counselling services and/or partner support groups.

## 5 CONCLUDING REMARKS

Achieving optimal recruitment rates can be difficult, particularly in relation to highly invasive trials. Our findings highlight critical stroke survivor and partner/carer concerns. Set against the context of cutting‐edge medicine and possible therapeutic misperception, clinical trial protocol requires the inclusion of fully embedded, multiple time point information, advice and support processes to ensure required care for stroke survivors and partners/carers throughout—and beyond—trial participation. Recruiter education training programmes should also ensure recruiters and health‐care professionals are alert to the individual information needs and support requirements of stroke survivors and partners/carers.

## 6 STRENGTHS AND LIMITATIONS

The use of Conversation Café created a relaxed, informal yet supportive environment to encourage discussion around this topic, creating rich data with the added bonus participants enjoyed the event. Some stroke survivors experienced moderate‐to‐severe aphasia, making prolonged speech tiring and full‐participation difficult. However, group organizers advised that the Café topic had generated such a high level of interest that some group members experiencing moderate‐to‐severe aphasia had spoken in public for the first time at this event.

Retrospectively, many participants revealed suggested cognitive and functional difficulties that could affect informed decision making. Nevertheless, retrospective questions concerning motivational, attitudinal, cognitive or affective states may also be affected by recall bias. Further, participants were not reflecting on actual trial participation. Therefore, views and motivations within a real setting are unknown.

Participants are not fully representative of the stroke survivor community. For example, Group A advised that while group numbers had significantly increased for the Conversation Café event, some people had advised they had chosen not to attend because they disagreed with research involving stem cells.
